# The influence of surrounding land cover on wetland habitat conditions: a case study of inland wetlands in South Korea

**DOI:** 10.7717/peerj.9101

**Published:** 2020-05-18

**Authors:** Ran-Young Im, Taekyu Kim, Chung-Yeol Baek, Chang-Su Lee, Song-Hyun Kim, Jung-Hwan Lee, Ji Yoon Kim, Gea-Jae Joo

**Affiliations:** 1 Department of Integrated Biological Sciences, Pusan National University, Busan, South Korea; 2 Global Environment Research Division, National Institute of Environmental Research, Incheon, South Korea; 3 Emission Inventroy Management Team, National Center for the Dust Information, Ministry of Environment, Cheongju, South Korea; 4 National Wetlands Center, National Institute of Environmental Research, Changnyeong, South Korea; 5 Academic Research Team, Gyeongsangnamdo Ramsar Environmental Foundation, Changnyeong, South Korea; 6 Wetland Research Team, National Institute of Ecology, Changnyeong, South Korea; 7 Nature and Ecology Policy Division, Nature Conservation Bureau, Ministry of Environment, Sejong, South Korea; 8 Division of Environmental Planning, Su Engineering Co. Ltd., Yangsan, South Korea; 9 Center for Climate Change Adaptation, National Institute for Environmental Studies, Tsukuba, Japan

**Keywords:** Catchment management, Rapid assessment, Spatial pattern, Wetland condition assessment, Wetland inventory

## Abstract

Wetland ecosystems have been globally degraded and lost due to rapid urbanization and climate change. An assessment of national scale inventory, including wetland types and conditions, is urgently required to understand the big picture of endangered wetlands, such as where they are and how they look like. We analyzed the spatial patterns of each inland wetland type (brackish wetland was included) in South Korea and the relative importance of land cover categories on wetland conditions. The wetlands were grouped into four dominant types (riverine, lake, mountain, and human-made) according to their topography. Riverine wetlands constituted the largest area (71.3%). The relative ratio of wetlands in a well-conserved condition (i.e., “A” rank) was highest in riverine wetlands (23.8%), followed by mountain wetlands (22.1%). The higher proportion of grasslands was related to a better condition ranking, but the increasing bareland area had a negative impact on wetland conditions. We also found that wetlands located near wetland protected areas tend to be in a better condition compared to remote sites. Our results further support the importance of the condition of surrounding areas for wetland conservation.

## Introduction

Globally, natural ecosystems are being converted for human use such as food production, road construction, and urban habitation (Foley, 2005). Human-induced land cover change is one of the most direct and influential factors resulting in the decline of biodiversity (Falcucci, Maiorano & Boitani, 2007; Newbold et al., 2015; Sala et al., 2000). Inland wetlands in freshwater ecosystems in particular have been rapidly degraded and connectivity among wetland habitats has decreased due to population growth and related anthropogenic development (Davidson, 2014; Gibbs, 2000; Mori, Onoda & Kayaba, 2018). Wetland habitat conditions are closely related to the species richness, functions, and associated services of wetland ecosystems (Engelhardt & Ritchie, 2001). Wetland biodiversity is further affected by land-use attributes around wetlands (Findlay & Bourdages, 2000; Houlahan et al., 2006), and surrounding areas also serve an important supporting function to wetlands (Mitsch & Gosselink, 2000). The benefits and positive functions of wetlands have been well recognized and the needs for their conservation have been highly suggested (Gibbons et al., 2006; Juffe-Bignoli et al., 2014; Maltby, 1991).

National wetland inventories are important as a first step to improve management plan and policy-making for wetland conservation; because they can provide an overview of the recent status including distribution, condition, and potential threats (Gardner & Finlayson, 2018; Hu et al., 2017; Kingsford, Basset & Jackson, 2016). To understand the status of wetland ecosystems, a wetland inventory has been carried on diverse spatial scales such as national, regional, and local (Davidson & Finlayson, 2007; Finlayson, Davidson & Stevenson, 2001; Finlayson & Van der Valk, 1995; Kloiber et al., 2015). Furthermore, diverse wetland assessments have been used to understand the ecological condition (Guidugli-Cook et al., 2017), functional value of wetlands (Fennessy, Jacobs & Kentula, 2007), ecosystem services (Costanza et al., 2007; Yang et al., 2008), and wetland health (Beuel et al., 2016). Although implementation of wetland inventory has been increased since 2000s, only 44% of the Ramsar Convention participants have yet to establish national inventories. The rate is higher in North America (67%) and Europe (62%), while in Asia region only 30% of participating countries established their national wetland inventory (Gardner & Finlayson, 2018).

The assessment of wetland condition or function (e.g., Hydrogeomorphic Wetland Classification; HGM or Rapid Assessment; RA) can be utilized for the effective management and conservation of wetland ecosystems (Fennessy, Jacobs & Kentula, 2007). There have been several trials of rapid assessment of wetlands in South Korea (Choi et al., 2017; Hong & Kim, 2017; Koo & Kim, 2001); however, nationwide assessment has been limited. For a comprehensive understanding of wetland condition in East-Asian region, it is necessary to organize national-level wetland inventory and information on current wetland condition in this region.

South Korea is one of the regions where land use is rapidly transforming, as it is elsewhere in East Asia, and wetland habitats are disappearing due to recent economic development. The agricultural conversion constituted the largest part of wetland loss during the 1930s in South Korea. Reclamations for the construction of roads and industrial complexes were rapidly increased in the late 1980s (Im et al., 2017). About 60% of floodplain wetlands developed in the lower Nakdong River disappeared in the last 90 years. The increasing pressure to modify aquatic infrastructure (i.e., levees, dams, channels, etc.) has threatened wetland ecosystems. In order to understand the distribution of wetlands and their general conditions, the Ministry of the Environment (MOE) and the National Institute of Environmental Research (NIER) conducted a wetland survey project since 2000. The MOE and NIER surveyed spatial information, type classification, and wetland condition. However, spatial patterns of wetland types, wetland conditions, and their relationships with the surrounding environments were not well understood. Considering the rate of urbanization and the loss of natural wetlands in recent decades, information on the spatial context of wetland distribution and the influence of land use on wetland condition is necessary to prioritize conservation management for different wetland types.

In this study, we identified (1) distribution and extent of wetland and (2) analyzed the spatial patterns of wetland types and (3) the relationship between wetland conditions and surrounding environments to identify factors that influence the condition of wetlands in South Korea. Our goal was to understand overall condition of different types of wetlands in the study region and compare the relative importance of landscape components on wetland condition. We utilized wetland condition rankings from the wetland inventory database established by the MOE and NIER. The relative influence of environmental variables including elevation, land cover (urban area, cropland, forest, grassland, bareland, and water), population density, distance from rivers, and distance from protected areas were explored to test the effect of the socio-physical environment on the habitat condition of various wetland types. We hypothesized the rank of wetland condition is closely related to the surrounding environmental conditions in watersheds.

## Materials & Methods

### Wetland database

We utilized nationwide survey records of inland wetlands organized by the MOE and NIER (NIER, 2018). This wetland database includes the field survey results of 2,499 sites from 2000 to 2015. The spatial coverage of the wetland database is the inland wetlands (brackish wetland was included) distributed in the national boundary of South Korea (N32°07′22″ ∼38°36 ′40″, E124°36′36″ ∼131°52′22″). We used wetland types, polygon data of wetland delineation, geographical coordinates (GPS point), and the wetland condition rankings of each wetland.

The MOE used a modified wetland type classification system of the Ramsar Convention to suit the wetland characteristic of South Korea (MOE, 2010). This classification system grouped various types of inland wetlands into four major categories (i.e., riverine, lake, mountain, and human-made). It includes 25 sub-wetland types based on the environmental characteristics of topography, soil, water sources, vegetation, and other hydrologic traits which are important for wetland formation and maintenance. However, some wetland types were only identified at limited sites and they share similar environmental settings with other wetland types. In this study, inland wetlands share characteristics at level IV (i.e., hydrology, soil, and vegetation), which were integrated and 15 sub-wetland types were used for the following analysis (Table 1).

**Table 1:** Classification of inland wetlands (brackish wetland was included) in South Korea. Inland wetlands were sub-categorized at four different levels based on the traits of topography, hydrology, soil, and vegetation.

**Level I**	**Level II** (topography)	**Level III** (water source)	**Level IV** (hydrology, soil, vegetation)	**Reference number**
Inland wetlands	Riverine	Brackish	Estuarine/deltas/salt marsh	R1
		Lotic zone	Rivers/streams/creeks	R2
		Lentic zone	Floodplain	R3
	Lake	Brackish	Lagoon	L4
			Reclaimed lake	L5
		Freshwater	Freshwater lake	L6
			Oxbow/dune slack	L7
	Mountain	Precipitation	Bog	M8
		Subsurface water	Fen	M9
		Subsurface /surface water	Marsh	M10
			Shrub dominant swamp /abandoned paddy field in high elevation area	M11
Human-made wetlands	–	Artificial lake	Artificial dam/reservoir	H12
		Agricultural inland fishery purpose	Rice paddy	H13
			Irrigation channel/fishing pond	H14
		Constructed	Retention pond/urban parks	H15

To define the wetland characteristics and classify types of wetlands, experienced wetland researchers, including two field crew members of geologists (or hydrologists) and botanists conducted field investigations from April 2000 to October 2015 (MOE, 2011). In the field, the survey team marked the potential boundary of a wetland on a printed aerial map based on vegetation and sediment conditions. GPS coordinates of extreme points of the boundary of the wetland were also recorded using handheld GPS devices (position accuracy: <1 m; AKN1MBT/GLONASS, Ascen GPS, ROK). Based on the field survey results, the GIS-team delineated the boundary of wetlands in the GIS database. This wetland DB targeted a wetland larger than 400 m^2^ that can be identified in aerial photographs at 1:5,000 scale. A georeferenced field drawing of the wetland boundary was used as a draft line for the following process. The MOE set the delineation guidelines for each wetland type at level II categories (Table 1; MOE, 2010). For example, the boundary of riverine- and lake-type wetlands was essentially set based on the sediment condition and development of the wetland plant communities. The outer boundary line was set to the highest water level during flooding or it was set to the line of the dike or levee if present. The delineation of mountain-type wetlands was primarily determined by the sub-catchment area and the development of wetland vegetation (i.e., *Alnus* spp., *Salix* spp., *Moliniopsis japonica* Hack., *Carex* spp.).

We further used the results of the wetland condition survey from the wetland database of the MOE, described in the previous section. The wetland condition was assessed by scoring the diverse aspect of site conditions using relative scale criteria. The assessment was composed of eight different categories (six common categories for all types of wetlands, and two specific categories for each type). The common categories include (1) wetland size, (2) representativeness of type characteristic, (3) level of physical modification, (4) academic or educational value (i.e., proximity to educational or research facilities, potential or current use as an ecological-learning site or ecological monitoring site, easy-accessibility for citizens), 5) diversity of wetland wildlife and presence of endangered species, and 6) invasion level of exotic species. For riverine-type wetlands, (1) periods of wetland formation (i.e., <1 year, 1–5, 5–10, 10–30, and ≥ 30 years) and (2) landscape value (i.e., characteristic riverine topography, point bar, oxbow, and meandering channel) were included in the evaluation criteria. For lake-type wetlands, (1) the ratio of aquatic vegetation in the open water area (i.e., <20%, 20–40%, 40–60%, 60–80%, and ≥ 80%) and (2) economic or cultural activities by local people (i.e., fishery, agricultural water, and historic sites) were included. For mountain-type wetlands, (1) the presence of peat layer and (2) soil saturation were included. Evaluation categories in wetland condition assessment were selected from that of a rapid assessment method modified by Koo & Kim (2001) and MOE (2011). The purpose of the assessment was to evaluate the priority of wetlands conservation and to determine the necessity for an intensive survey to proceed with the designation of a protected area (MOE, 2011).

Each evaluation category was scored using five-point Likert scales (Tam et al., 2017). A higher score (five point) was related to the better condition of wetland environments. Each wetland site was classified into four ranking groups based on the average score of eight evaluation categories (Table 2). By using raw scoring data for wetland conditions from 2000 to 2010 (during the first 10 years), the MOE tested the distribution of total scores and defined criteria of four ranks as described in Table 2. Rank “A” represents wetlands in a well-conserved condition, which had the highest score with more than half of the evaluation criteria. Rank “B” includes wetlands with an average score higher than 2.8. Rank “C” indicates wetlands that require moderate enhancement to recover to a healthy condition. Sites with an average value of less than 2.0 were evaluated with rank “D” where sites were severely modified and active restoration practices would be required to recover its previous status.

**Table 2:** A judgment criteria for an assessment of wetland condition rankings. The assessment comprised eight categories and was scored using five-point Likert scales.

**Rank**	**Criteria**	**Conservation priority**
A	⋅ Score of “5” is more than 1/2 of the total assessment criteria ⋅ Scores of type-specific criteria are both “5” ⋅ Important habitat for endangered wildlife, core habitat of legally protected species	Absolute conservation
B	⋅ The average of total score: ≥ 2.8 ⋅ Score of “5” is more than 1/4 of the total assessment criteria ⋅ Score of “4” is more than 1/2 of the total assessment criteria ⋅ Scores of type-specific criteria are both >4 ⋅ Sites fulfill the requirement for an “A” ranking, but had temporal (not permanent) degradation or damage	Conservation
C	⋅ The average of total score: 2.0 ∼2.7 ⋅ At least one criteria is scored “5” ⋅ Score of “3″is more than 1/2 of the total assessment criteria	Conservation and wise usage
D	⋅ The average of total score: <2.0 ⋅ Sites have been permanently damaged or undergone severe modification	Restoration or use

### Wetland distribution analysis

We calculated the central point and size of wetlands by using the polygon data of the wetland delineation database. These vector data of each wetland were used to extract the spatial pattern in the following analysis. To understand the distribution pattern of wetland condition rankings, we applied a kernel density function and calculated the relative frequency of the wetland condition ranking (%) in a catchment area by using ArcMap (v 10.1, ESRI, USA).

### Analysis of factors affecting wetland rankings

The associations between wetland condition rankings (A–D) and environmental variables were analyzed with multinomial regression models. Previous studies reported the significant influence of watershed environments compared to the influence of a distance-based buffered area (Croft-White et al., 2017). We used a catchment polygon from the Korean Reach File version 3.0 produced by the Water Information System of the MOE (http://water.nier.go.kr/front/riverNetwork/riverNetwork.jsp). This catchment DB divided the extent of our study sites into 117 sub-level catchments.

We considered environment variables such as elevation, the relative percentage of land cover class, population, distance from a river, and distance from a wetland protected area designated by MOE as explanatory variables. The elevation (El. m) of each wetland was extracted from the digital elevation model produced by the National Geographic Information Institute (https://www.ngii.go.kr/eng/main.do). The distance from the river and wetland protected area was calculated using the Euclidian distance function of the spatial analysis tool. We used log-transformed values for elevation, distance to the river and distance to the wetland protected area. The land cover data used a 1:50,000 scale map classified into seven categories (urban area, cropland, forest, grassland, wetland, bareland, and water) published by the MOE in 2000. The relative percentage of surrounding land cover (%) and population density (individuals per km^2^) was calculated by using a spatial analysis tool based on the catchment area of each wetland.

Before the regression analysis was conducted, the multicollinearity among variables was determined by calculating the variance inflation factors (VIF). Variables with <10 VIF remained in the analysis (Table S1). The regression models were run separately for different wetland types. We reported exp (*β*) (odds ratio), 95% confidence intervals (CI) for exp (*β*), *p*-values, and *R*^2^ (Nagelkerke’s) of the final regression model. An analysis of the multinomial regression and variance inflation factors were performed using IBM SPSS statistics software (version 20, IBM Corp., Armonk, NY, USA).

## Results

### Spatial patterns of wetlands

**Figure 1:**
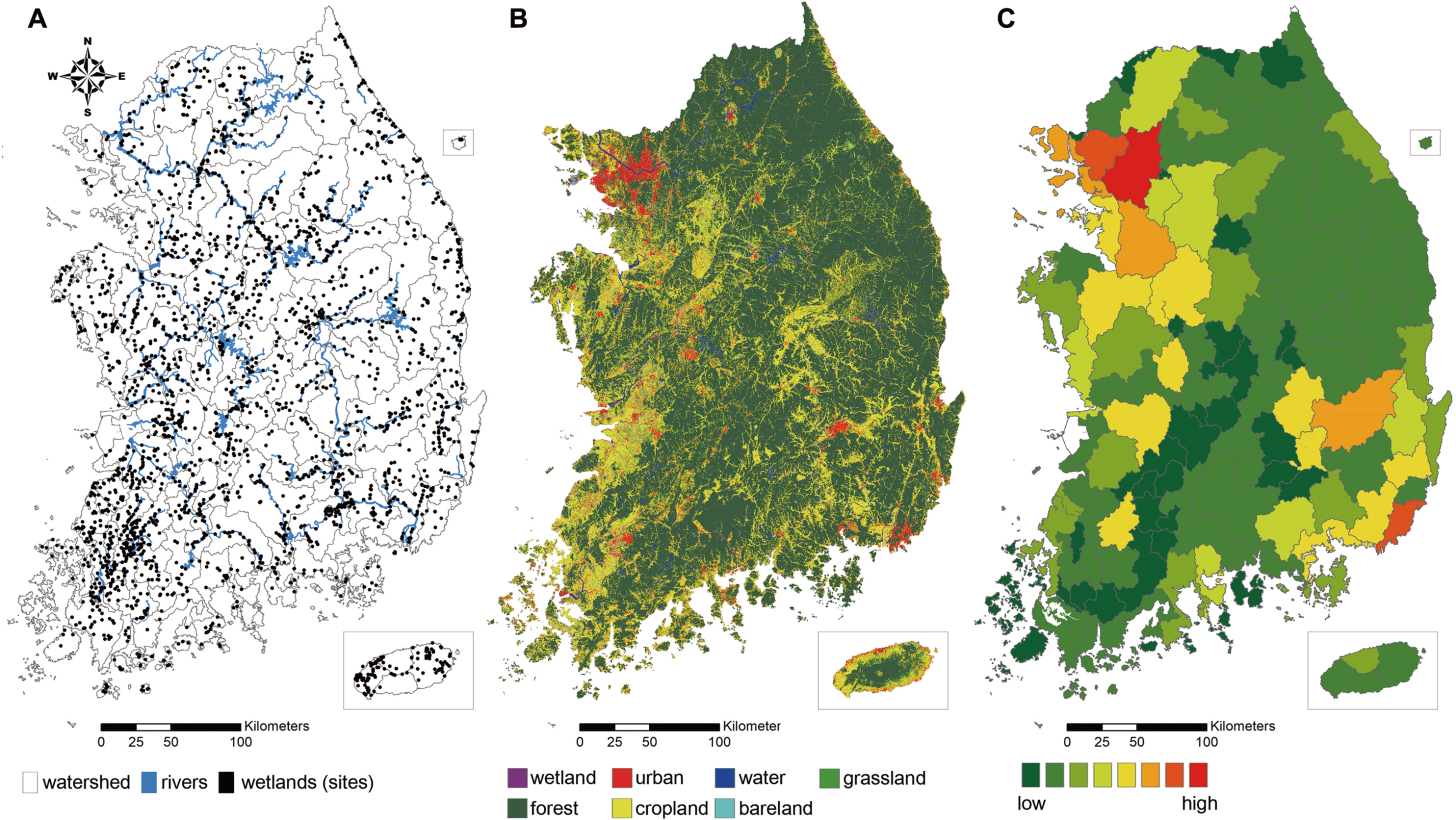
Study site and spatial pattern of land use characteristics of the extent of the study. (A) Distribution map of wetlands surveyed from 2000 to 2015 in South Korea; 2,499 wetlands polygons were represented in black, river channels in blue, grey lines represent the boundaries of catchments. (B) Land cover map with 1:50,000 scale classified into seven categories (urban area, cropland, forest, grassland, wetland, bareland, and water). (C) Population density in a catchment area.

The sum of the identified wetland area was 734.4 km^2^ and the size largely varied from 0.05 km^2^ to 58 km^2^ (Fig. 1A). A large portion of the wetlands (about 90% of the total) was distributed at a low elevation (<150 El. m). Regionally, the overall density of wetlands was higher in the western part of the Korean Peninsula, where the elevation was lower and agricultural intensity was higher (Fig. 1B). Three major river channels also run through the western region. The southeastern part, a tributary of the Nakdong River, also has a high density of wetlands sites. Near the metropolitan cities, the number of remaining wetlands was relatively low compared to rural areas (Fig. 1C).

**Figure 2:**
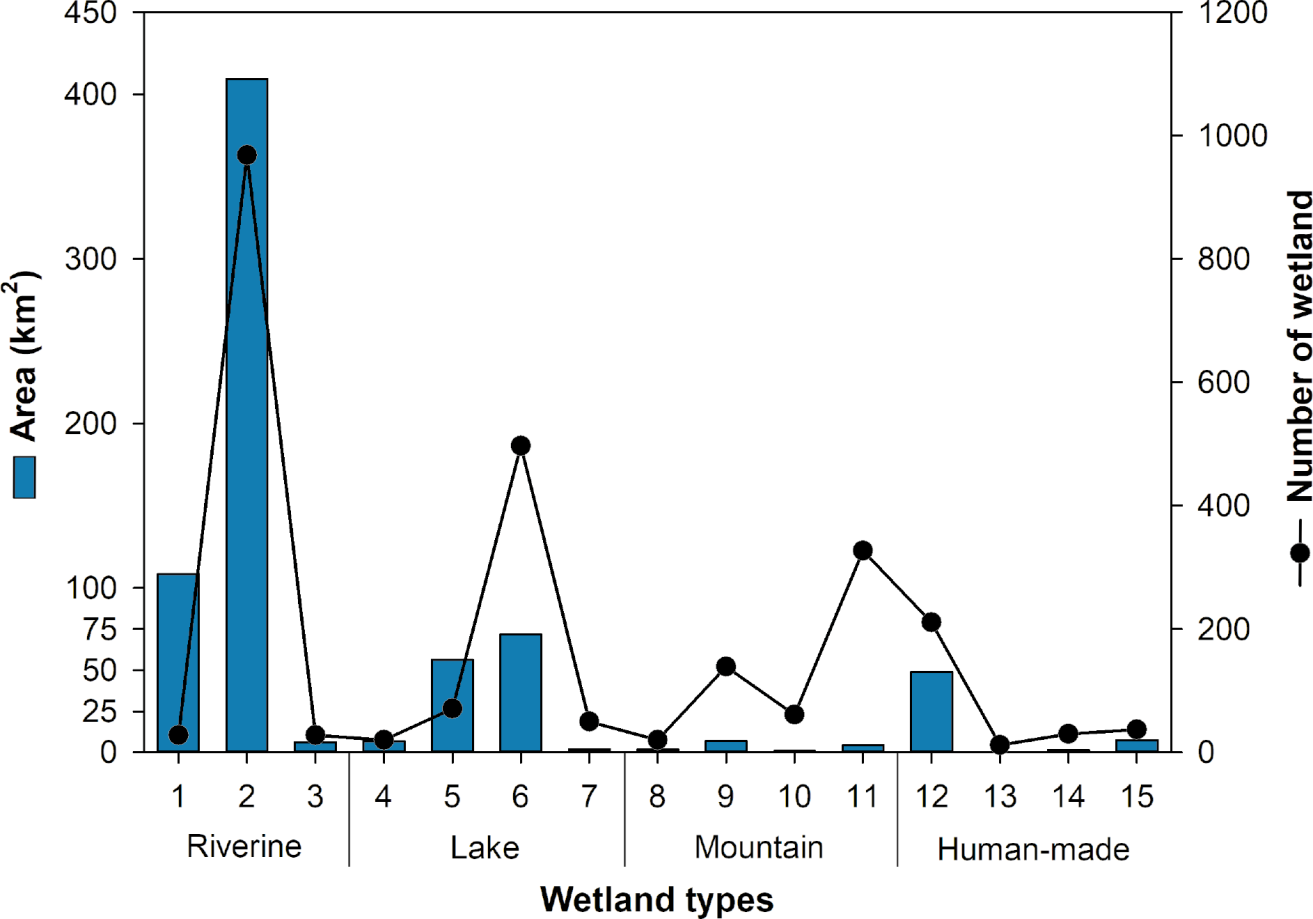
Area and number of wetlands with different wetland types. More details on wetland types can be found in Table 1. The wetland area was represented with a bar graph in blue and the number of wetlands was represented by the line graph and points in black.

Riverine-type wetlands had the largest area (524.0 km^2^, 71.3% of the total), followed by lake (137.1 km^2^, 18.7%), human-made (58.6 km^2^, 8.0%), and mountain types (14.6 km^2^, 2.0%; Fig. 2). Lotic-type wetlands (R2) constituted the largest portion of riverine-type wetlands (409.3 km^2^, 968 sites), followed by brackish- (R1; 108.5 km^2^, 28 sites) and lentic-type wetlands (R3; 6.2 km^2^, 28 sites). Freshwater lakes (L6) had the largest portion among the lake-type wetlands (71.8 km^2^, 497 sites), followed by reclaimed lakes (L5; 56.4 km^2^, 71 sites), lagoons (L4; 6.8 km^2^, 20 sites), oxbow or dune slacks (L7; 2.1 km^2^, 50 sites). The average size of reclaimed lakes was notably higher than other wetland types. Artificial dams or reservoirs (H12; 49.0 km^2^, 211 sites) constituted the largest portion of human-made wetland types. Retention ponds or urban parks (H15) covered 7.6 km^2^ (37 sites). Irrigation channels or fishing ponds (H14) and rice paddy wetlands (H13) covered 1.4 km^2^ (30 sites) and 0.7 km^2^ (12 sites), respectively. Fens (M9; 7.1 km^2^, 139 sites) were the dominant type of mountain wetland, followed by shrub dominant swamp or abandoned paddy fields (M11; 4.5 km^2^, 327 sites), bogs (M8; 2.0 km^2^, 20 sites), and marshes (M10; 0.9 km^2^, 61 sites).

### Assessment of wetland conditions

**Figure 3:**
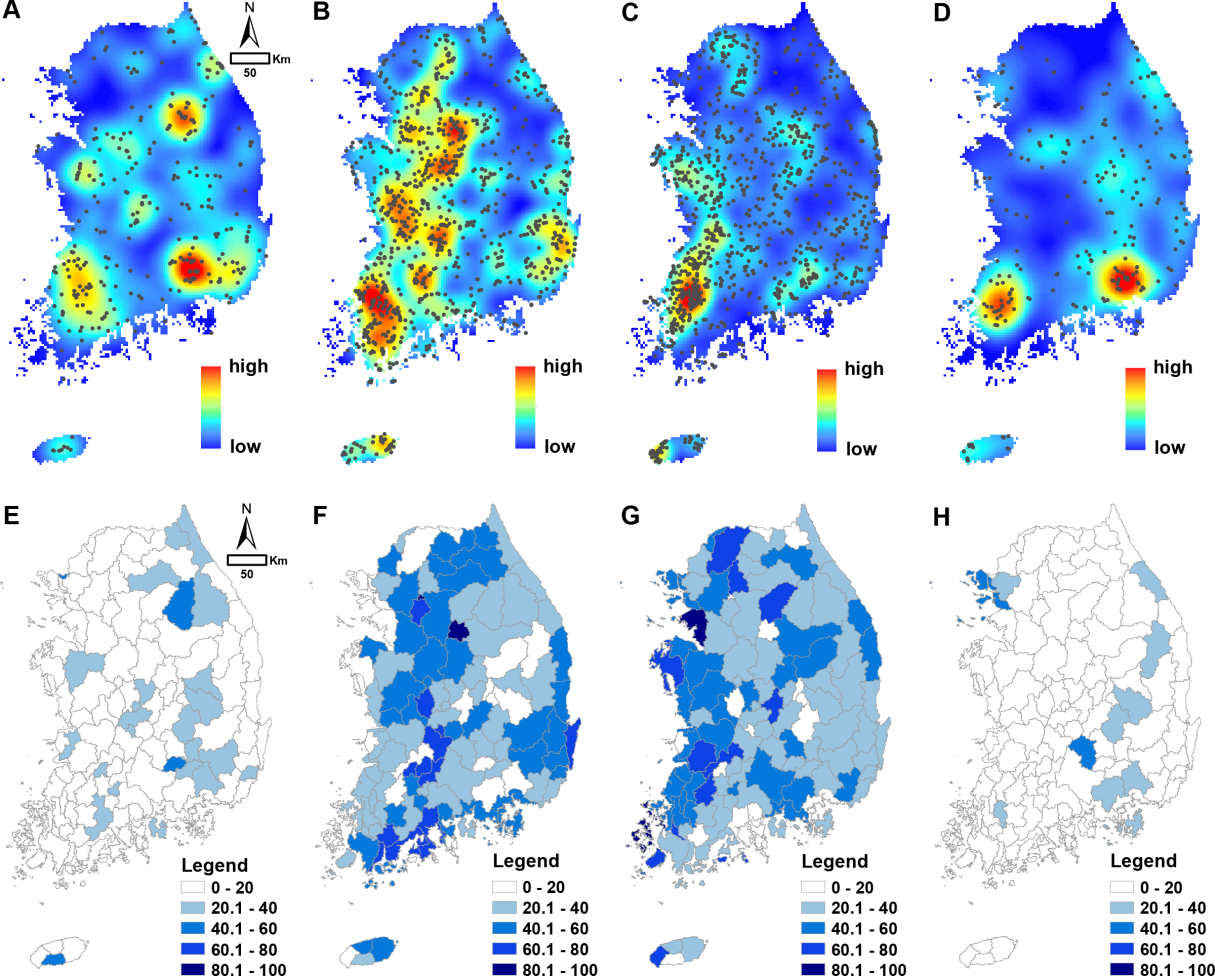
Spatial pattern of wetland condition rankings. (A–D): point density of wetlands with different ranks (A: A rank; B: B rank; C: C rank; D: D rank); (E–H): relative frequency of wetland rankings (%) in the catchment area (E: A rank; F: B rank; G: C rank; H: D rank).

Based on the relative evaluation criteria of wetland conditions, we assessed 2,497 wetland sites out of 2,499 sites identified from the previous survey (two sets of missing data). There was a total of 303 “A”-ranked wetlands (12.1% of total; Figs. 3A and 3E), which represent those in a well-conserved condition. “A”-ranked wetlands were largely distributed near the wetland protected area in the northern (e.g., Hanbando wetland) and southern (e.g., Upo wetland) part. “B”- and “C”-ranked wetlands made up 938 (37.6%) and 1,039 sites (41.6%; Figs. 3B–3C, 3F–3G), respectively. “B”- and “C”-ranked wetlands were distributed broadly in the western part of the peninsula, where the relative percentage of agricultural fields is high. “D”-ranked wetlands, those that are in a severely modified or contaminated state, made up a total of 217 sites (8.7%; Figs. 3D and 3H). “D”-ranked wetlands showed a high level of density in the two rural areas in the southern region. These areas were adjacent to the industrial complex. The density of “D”-ranked wetlands was higher in the metropolitan area which is related to a high population density (Fig. 3H). Comparisons among different wetland types identify distinct spatial patterns related to its origin and potential influence of urbanization (Figs. S1–S4). “A”-ranked riverine wetland and mountain wetland largely distributed in the upstream area and high elevated mountainous region, respectively. Regardless of wetland types, “D”-ranked wetland sites tended to distribute in urban areas or in suburban areas, where urban development was high.

**Figure 4:**
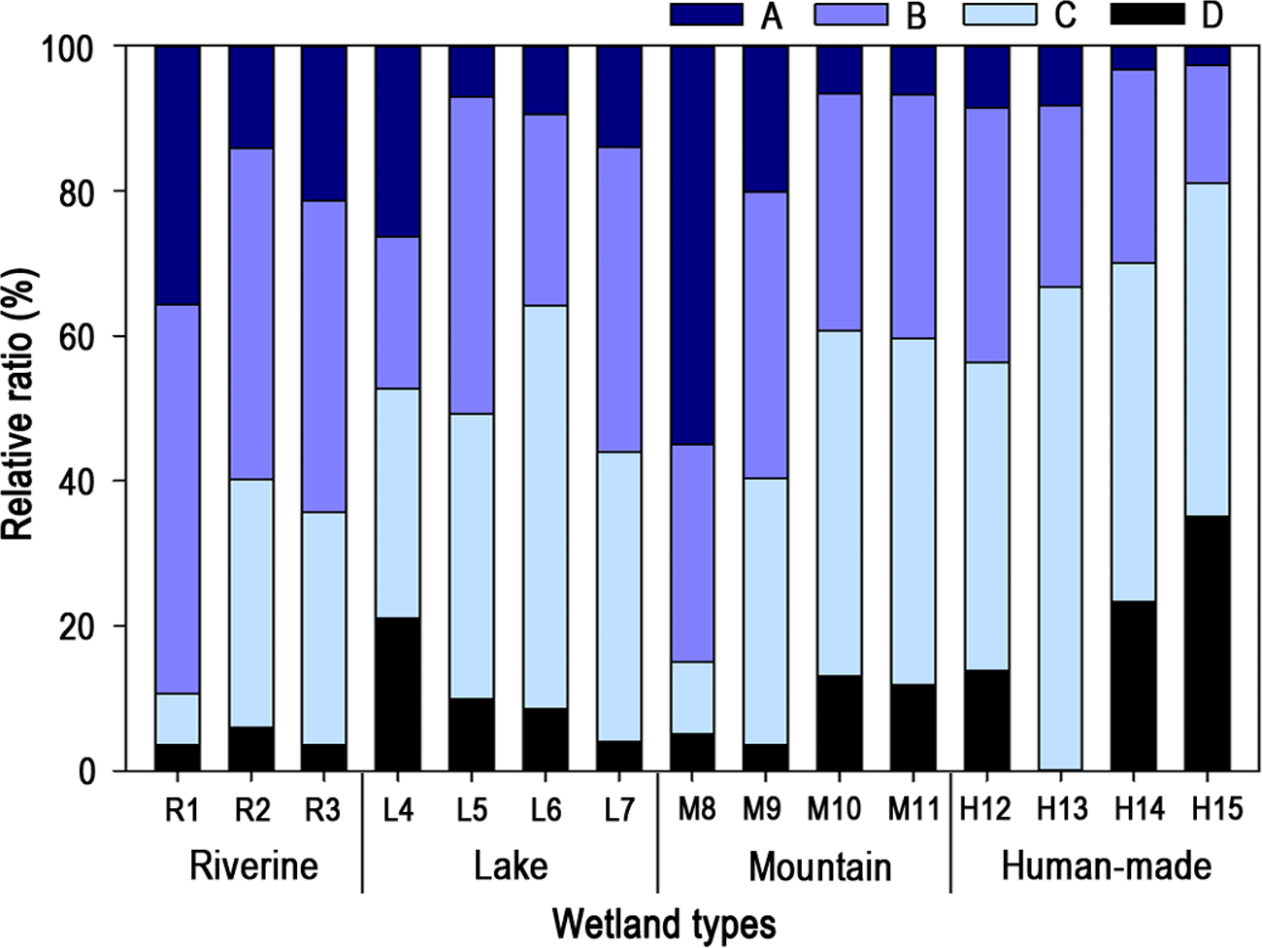
Relative frequency (%) of wetland condition rankings (A–D) by various types of wetlands in South Korea. More details on wetland types can be found in Table 1.

The relative ratio of “A”-ranked wetlands was highest in the riverine type (23.8%), followed by mountain (22.1%), lake (14.2%), and human-made (5.7%) wetlands (Fig. 4). Bog (M8; 55.0%) showed the highest rate for “A”-ranked wetlands. brackish wetlands (R1; 35.7%) and lagoons (L4; 25.5%) also showed relatively good condition compared to other wetland types. Human-made wetland types recorded the lowest ratio for the higher two ranks and the highest ratio for the lower ranks. The relative ratio of “D”-ranked wetlands was highest in the human-made-type (18.1%), followed by lake (10.8%), mountain (8.4%), and riverine wetlands (4.4%).

### Factors affecting wetland condition rankings

The regression model for riverine-type wetlands had a significant effect relative to the area of grassland (%) and bareland (%) in the watershed and distance from a wetland protected area (*R^2^* = 0.16, *N* = 1023, *P* < 0.001; Table 3). The area of grasslands in the watershed was about 1.67 (“A”-ranked), 1.91 (“B”-ranked), and 1.66 (“C”-ranked) times higher than “D”-ranked sites (*P* < 0.001); however, the area of bareland was 0.47 (“B”-ranked) and 0.62 (“C”-ranked) times lower in the riverine wetlands. Riverine wetlands located near the protected area in the watershed were tended to have a higher condition ranking. The rank of lake-type wetlands showed significant effects relating to the area of grassland and bareland (*R^2^* =** 0.17, *N* = 631, *P* < 0.001; Table 3). “B”-ranked lake-type wetlands had grassland areas that were 1.26 times larger in their watershed than “D”-ranked lakes. And the area of bareland of “C”-ranked lake-type wetlands was about 1.37 times larger than “D”-ranked sites. Elevation and distance to protected area affected the condition of the mountain-type wetlands (*R^2^* = 0.19, *N* = 547, *P* < 0.001). Wetlands located at a high elevation (∼1,216 m) tended to have a higher condition ranking. “A”-ranked mountain-type wetlands were located close distance to the protected area than “D”-ranked sites. Human-made wetlands with larger water and grassland areas in their watershed tend to have a better wetland condition.

**Table 3:** Multinomial logistic regression model of wetland condition rankings with characteristics of surrounding environments. Models were run separately with different wetlands types, and rank “D” was used as the reference category. Exp(*β*) means odd ratio and how many times the value of explanatory variables increases compared to that of “D” rank.

**Rank**	**Variables**	**Riverine (*n* = 1,023)**		**Lake (*n* = 631)**		**Mountain (*n* = 547)**		**Human-made (*n* = 290)**	
		Exp(*β*)	95% CI	Exp(*β*)	95% CI	Exp(*β*)	95% CI	Exp(*β*)	95% CI
**A**	Elevation	0.71	0.33, 1.51	0.80	0.40, 1.57	3.74 ^**^	**1.38, 10.10**	1.36	0.41, 4.50
	Agriculture	0.99	0.97, 1.02	0.99	0.96, 1.02	0.98	0.95, 1.02	1.00	0.96, 1.05
	Bareland	0.69	0.43, 1.13	1.47	0.98, 2.19	1.08	0.85, 1.38	0.56	0.28, 1.13
	Forest	1.00	0.95, 1.05	1.00	0.96, 1.05	0.99	0.94, 1.05	0.97	0.92, 1.03
	Grassland	1.67 ^***^	**1.34, 2.08**	1.14	0.94, 1.39	1.21	0.95, 1.55	1.17	0.85, 1.62
	Urbanization	0.91	0.80, 1.03	0.77	0.58, 1.02	1.00	0.72, 1.39	0.99	0.70, 1.42
	Water	0.91	0.73, 1.14	1.39	0.86, 2.25	0.96	0.66, 1.39	1.55 ^*^	**1.02, 2.35**
	Distance to protected area	0.48 ^*^	**0.26, 0.89**	0.51	0.26, 1.01	0.62 ^*^	**0.40, 0.97**	0.80	0.35, 1.84
	Distance to river	1.01	0.77, 1.34	1.22	0.83, 1.79	1.11	0.54, 2.27	0.97	0.64, 1.46
	Population	0.99	0.99, 1.01	1.02	0.99, 1.04	0.99	0.97, 1.01	0.98	0.95, 1.02
**B**	Elevation	0.56	0.28, 1.10	0.66	0.38, 1.17	2.25 ^*^	**1.13, 4.49**	0.91	0.43, 1.94
	Agriculture	1.00	0.98, 1.03	0.99	0.96, 1.01	1.01	0.98, 1.04	1.00	0.97, 1.04
	Bareland	0.47 ^**^	**0.30, 0.73**	1.21	0.88, 1.66	1.08	0.85, 1.38	1.24	0.78, 1.96
	Forest	1.01	0.97, 1.06	1.02	0.98, 1.06	1.00	0.96, 1.05	1.03	0.99, 1.08
	Grassland	1.91 ^***^	**1.56, 2.34**	1.26 ^**^	**1.08, 1.47**	1.16	0.94, 1.42	1.31 ^*^	**1.05, 1.64**
	Urbanization	1.01	0.97, 1.06	0.99	0.79, 1.24	1.12	0.87, 1.44	1.04	0.85, 1.28
	Water	0.89	0.74, 1.07	1.49	0.98, 2.27	1.04	0.78, 1.39	1.28	0.89, 1.85
	Distance to protected area	1.25	0.67, 2.35	1.38	0.67, 2.87	1.62	0.97, 2.72	1.23	0.60, 2.53
	Distance to river	1.02	0.79, 1.31	1.08	0.80, 1.46	1.69	0.90, 3.16	1.27	0.95, 1.70
	Population	0.99	0.99, 1.01	1.00	0.99, 1.02	0.99	0.98, 1.01	1.00	0.98, 1.02
**C**	Elevation	1.05	0.52, 2.12	1.20	0.70, 2.08	1.32	0.68, 2.55	0.89	0.45, 1.77
	Agriculture	1.01	0.99, 1.04	1.00	0.98, 1.03	0.99	0.96, 1.02	0.99	0.97, 1.03
	Bareland	0.62 ^*^	**0.40, 0.96**	1.37 ^*^	**1.01, 1.85**	1.08	0.85, 1.38	1.10	0.72, 1.69
	Forest	1.00	0.96, 1.05	1.03	0.99, 1.07	1.00	0.96, 1.04	1.02	0.98, 1.06
	Grassland	1.66 ^***^	**1.36, 2.03**	1.03	0.89, 1.20	1.16	0.95, 1.42	1.10	0.89, 1.37
	Urbanization	1.02	0.98, 1.07	1.17	0.94, 1.44	1.13	0.88, 1.45	1.12	0.93, 1.35
	Water	0.99	0.84, 1.18	1.41	0.93, 2.14	1.09	0.82, 1.45	1.08	0.75, 1.54
	Distance to protected area	1.16	0.61, 2.21	1.10	0.57, 2.13	1.35	0.84, 2.17	1.39	0.72, 2.68
	Distance to river	1.05	0.82, 1.36	1.20	0.90, 1.60	0.95	0.54, 1.67	1.21	0.93, 1.59
	Population	0.99	0.98, 1.00	0.99	0.97, 1.00	0.99	0.98, 1.01	0.99	0.97, 1.01
Nagelkerke *R*^2^		0.16		0.17		0.19		0.15	
*χ*^2^		159.62		105.47		104.27		43.15	
*P*		<0.001		<0.001		<0.001		0.057	

**Notes.**

*** *P* < 0.001.

** *P* < 0.01.

* *P* < 0.05.

Values in bold letters show significant correlations (*p* <0.05).

## Discussion

### Current condition of wetlands in South Korea

Considering the number and area of wetlands, riverine-type wetland was the most dominant type (e.g., 71.3% of the total number) in the study region. River channels (55.7%) and brackish wetlands (14.8%) constituted the biggest portion of riverine-type wetlands. On the other hand, the relative number of floodplain wetlands in the riverine wetlands was low in our results (0.8%), even though it is known as one of the highest value ecosystems on earth (Costanza et al., 1997). Floodplain wetlands have decreased globally (e.g., in Europe and North America: up to a 90% loss; Australia: about a 50% loss), and more than half of the area was lost due to conversion to agricultural fields and dam construction (Kingsford, 2000; Tockner & Stanford, 2002). In South Korea, most of the floodplain wetlands were lost due to the construction of levees at a high elevation and the conversion of land to agricultural fields (Im et al., 2017). Recently, sixteen large weirs were newly built in the four major rivers and floodplains, further modifying the hydrologic condition of major river systems (Im et al., 2015).

Artificial wetlands (i.e., reservoirs, dams, lakes, rice paddies, artificial waterways, water purification ponds, etc.) were rated with the highest percentage of the lowest rank (C and D rank: 68.6%), reflecting their degraded habitat condition compared to other wetland types. Most of the artificial wetlands were located close to cities or industrial complexes where anthropogenic activities were higher (Wu, Zhou & Tian, 2017). Intense land use, pollution, and the drainage of water would have complexly influenced the condition of artificial wetlands. However, the number of these human-made wetlands has been increasing (Finlayson & Spiers, 1999) and the importance of well-designed wetlands with proper management reflect the significant value of the wildlife that reside there as well as water quality improvement (Ghermandi et al., 2010). A detailed survey of the conservation status of human-made wetlands is required and adequate restoration or management plans need to focus on increasing the health of human-made wetlands.

Agricultural land occupied about 18.8% (rice paddies 11.2%, 11,282.1 km^2^) of South Korea (KOSIS, 2018). Rice paddies were included in the category of wetland types; however, only part of the rice paddies managed by the national trust and abandoned fields were included in the current wetland inventory due to practical management reasons. In contrast with the US and Europe, rice paddies in Asia occupy a larger portion of the agricultural land area than dry crop fields. The diverse function of the rice paddy system, such as flood control, groundwater recharge, water quality, local climate mitigation, biodiversity, and culture and landscape has been supported by various studies (Natuhara, 2013; Settele et al., 2018). Contracting parties of the Ramsar Convention adopted the “Enhancing biodiversity in rice paddies as wetland systems” resolution during the 10th Ramsar Convention (2008). Organic farming, winter flooding, and Biodiversity Management Contracts further enhanced the ecological function of rice paddy ecosystems. Considering the diverse benefits of rice ecosystem services in Asia, following wetland evaluation systems need to reflect the distinct characteristics of the rice paddy system.

### Importance of surrounding land cover in catchments

We found (1) the positive influence of grassland, elevation, and protected areas on wetland conditions and (2) the negative influence of bareland in the catchment area. Landscape modification and pollution induced by human society have been steadily increasing and it has caused the massive loss of wetland ecosystems (Beuel et al., 2016; Gibbs, 2000). Depending on the types of wetlands, the land use pattern influences the condition of wetlands in various ways (Vörösmarty et al., 2010). Other studies showed that the proportion (%) of agricultural fields (Heady et al., 2015; Stapanian, Gara & Schumacher, 2018) and urban areas (Haidary et al., 2013; Houlahan & Findlay, 2003) in surrounding areas negatively affected the biological conditions of wetlands. Forests had a positive influence on mitigating water quality degradation (Sliva & Williams, 2001) and maintaining biodiversity (Houlahan et al., 2006) and the quality of wetland vegetation (Stapanian, Gara & Schumacher, 2018). Our results showed that the high frequency (%) of grasslands in the catchment area positively affected the condition rank of wetlands regardless of wetland type. Grassland with a dense vegetation cover has higher soil retention capacity (Fu et al., 2011). Thus, its soil erosion control service prevents sediment inflow into rivers or wetlands in the catchment area and helps to improve water quality (Brazier, Bilotta & Haygarth, 2007). Besides, grassland around wetlands can provide more natural habitats with a synergistic effect for enhanced biodiversity (Naugle et al., 2001). Overall, the results imply the importance of natural vegetation in the catchment area (Bengtsson et al., 2019; Smith & Haukos, 2002). On the other hand, the high proportion of bareland (i.e., areas with no vegetation and bare soil) negatively influenced wetland condition rankings. Bareland is known to have higher soil erosion rates than other types of land cover (Cerdan et al., 2010), and the sediment flows into the wetland increases internal turbidity and reduces light transmission (Wantzen & Mol, 2013). Besides, dormant eggs (e.g., invertebrate) or seeds can be buried by sedimentation resulting in reduced survival rate; a higher rate of soil erosion can negatively affect the sustainability of wildlife in wetlands (Gleason et al., 2003). Considering various land use factors (i.e., agriculture, urban areas, forest, and vegetation) related to wetland conditions, it seems that the significant land use factors affecting wetland conditions can be diverse depending on the landscape characteristics of the catchment area and wetland types.

The proximity to the protected wetland area notably showed a positive influence on the condition ranking of riverine and mountain-type wetlands. Barber et al. (2014) reported that protected areas had the effect of mitigating disturbances even in the surrounding areas. McDonald et al. (2007) reported that the rate of land conservation increased within 2 km of the protected area, and the newly established protected area tended to be located close to previous protected areas. The positive adjacent influence of protected areas on the surrounding area needs to be concurrently considered as synergistic with regional conservation practices (Beatty et al., 2014).

### Recommendations for wetland management

The necessity of wetland inventory has become important in providing effective conservation plans. We showed that an evaluation of wetland conditions with relative scoring can be used to make the protection or rehabilitation area a priority in order to enhance the condition of degraded wetlands. However, there has been a gap in the available quantitative information due to short surveys and lack of clarity in the evaluation of wetland condition. Majority of academic surveys have been spatially biased in conservation areas or proximate sites from research institutes. The absence of quantitative measurements in most wetland sites limits a detailed evaluation of wetland conditions. To compensate for this issue, we suggest the utilization of historical literature, including NGO surveys, academic reports, and land use records from the past. There were several successful attempts to integrate and mine new information by using this historical information (Griffin, 2017; Kim, Joo & Do, 2018), and these practices would be beneficial to compensate for the results of the initial assessment.

Wetland conditions are sensitive to direct environmental change (i.e., water quality, reclamation) within the wetlands, and can also be negatively affected by land cover changes in surrounding areas (Stapanian, Gara & Schumacher, 2018). Current studies supported the direct and indirect influence of land use changes in the watershed (Croft-White et al., 2017). Richardson et al. (2011) found that upstream restoration efforts had a positive influence on the downstream water quality of rivers and wetlands. Our results further stress the importance of an integrated watershed approach for the successful management and restoration of wetlands.

In addition, our results showed that five of the wetland types (i.e., brackish wetlands, riverine, floodplains, bog, and fen) are maintaining better conditions (% of “A+B”-ranked: 60–89%) compared to other wetland types. Freshwater lake, marsh, and abandoned paddy field in high elevation area had a higher portion of degraded wetland sites (“C+D” ranked: 60–64%). Conservation management should consider this biased situation among different types of wetlands. And conservation efforts to improve surrounding land use conditions with a natural area (e.g., grassland) are required to support wetland conditions. Finally, expanding and maintaining wetland conservation areas could enhance the overall condition by strengthening habitat networks in the watershed.

## Conclusions

Establishing national wetland inventory and identifying the spatial pattern of wetland condition is important for effective wetland conservation. In this study, we mapped the inland wetlands in South Korea and evaluated the overall condition of wetlands based on the rapid assessment method. We expect our results will provide a rough estimation of the wetland status in the study region and can be a basis to plan effective conservation schemes. We also found that wetland condition was influenced by the land cover condition in sub-watersheds. Based on this finding, we stress the importance of considering land cover characteristics in the watershed to protect diverse types of wetland habitats.

##  Supplemental Information

10.7717/peerj.9101/supp-1 Data S1 The result of variance inflation factors (VIF) of environmental variables

10.7717/peerj.9101/supp-2 Figure S1 Spatial pattern of wetland condition ranking of riverine-type wetlands (A)–(D): point density of riverine-type wetlands with different ranks (A: A rank; B: B rank; C: C rank; D: D rank); (E)–(H): relative frequency of riverine-type wetland rankings (%) in the catchment area (E: A rank; F: B rank; G: C rank; H: D rank)

10.7717/peerj.9101/supp-3 Figure S2 Spatial pattern of wetland condition ranking of lake-type wetlands (A)–(D): point density lake-type wetlands with different ranks (A: A rank; B: B rank; C: C rank; D: D rank); (E)–(H): relative frequency of lake-type wetland rankings (%) in the catchment area (E: A rank; F: B rank; G: C rank; H: D rank)

10.7717/peerj.9101/supp-4 Figure S3 Spatial pattern of wetland condition ranking of mountain-type wetlands (A)–(D): point density of mountain-type wetlands with different ranks (A: A rank; B: B rank; C: C rank; D: D rank); (E)–(H): relative frequency of mountain-type wetland rankings (%) in the catchment area (E: A rank; F: B rank; G: C rank; H: D rank)

10.7717/peerj.9101/supp-5 Figure S4 Spatial pattern of wetland condition ranking of human-made wetlands (A)–(D): point density of human-made wetlands with different ranks (A: A rank; B: B rank; C: C rank; D: D rank); (E)–(H): relative frequency of human-made wetland rankings (%) in the catchment area (E: A rank; F: B rank; G: C rank; H: D rank)

## Additional Information and Declarations

### Data Availability

The following information was supplied regarding data availability: The wetland datasets are available at the Open Data Portal of South Korea (in Korean): https://www.data.go.kr/dataset/3038409/fileData.do.
