# Supplementary material for: The influence of surrounding land cover on wetland habitat conditions: a case study of inland wetlands in South Korea
Source: PeerJ. 2020 May 18;8:e9101. doi: 10.7717/peerj.9101 (PMC7241414; doi:10.7717/peerj.9101)
Supplement: Data S1 [file peerj-08-9101-s001.docx]

**Supplemental_data_S1**

Table. S1. The result of variance inflation factors (VIF) of environmental variables.

| **Environmental variables** | **VIF** |
| --- | --- |
| Elevation | 1.41 |
| Agriculture | 1.91 |
| Bareland | 2.37 |
| Forest | 5.41 |
| Grassland | 1.64 |
| Urbanization | 6.21 |
| Water | 1.13 |
| Distance to protected area | 1.08 |
| Distance to river | 1.08 |
| Population | 3.80 |
