# Supplementary figures and images for: The influence of surrounding land cover on wetland habitat conditions: a case study of inland wetlands in South Korea
Source: PeerJ. 2020 May 18;8:e9101. doi: 10.7717/peerj.9101 (PMC7241414; doi:10.7717/peerj.9101)

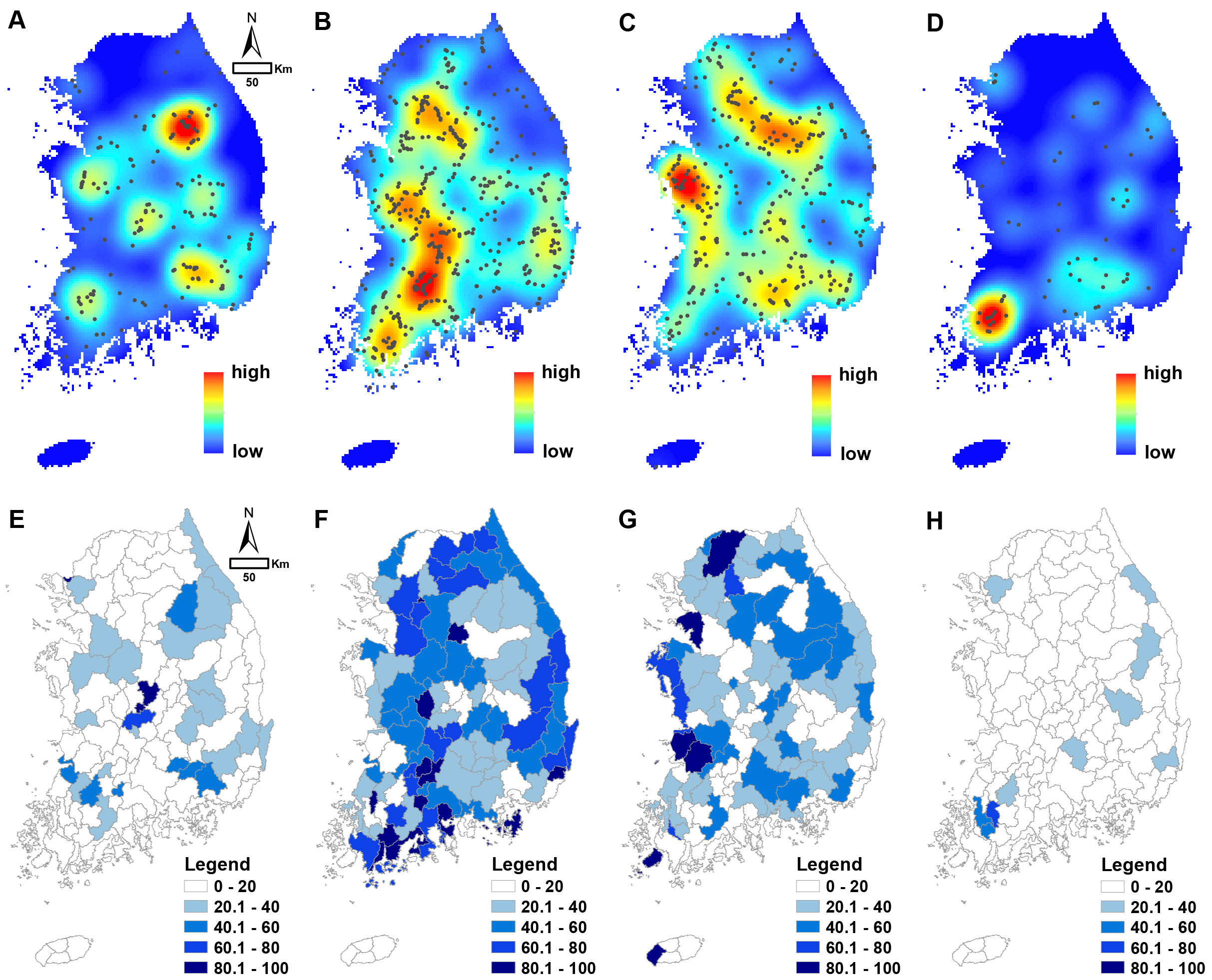

Supplement: Figure S1 — (A)–(D): point density of riverine-type wetlands with different ranks (A: A rank; B: B rank; C: C rank; D: D rank); (E)–(H): relative frequency of riverine-type wetland rankings (%) in the catchment area (E: A rank; F: B rank; G: C rank; H: D rank) [file peerj-08-9101-s002.png]

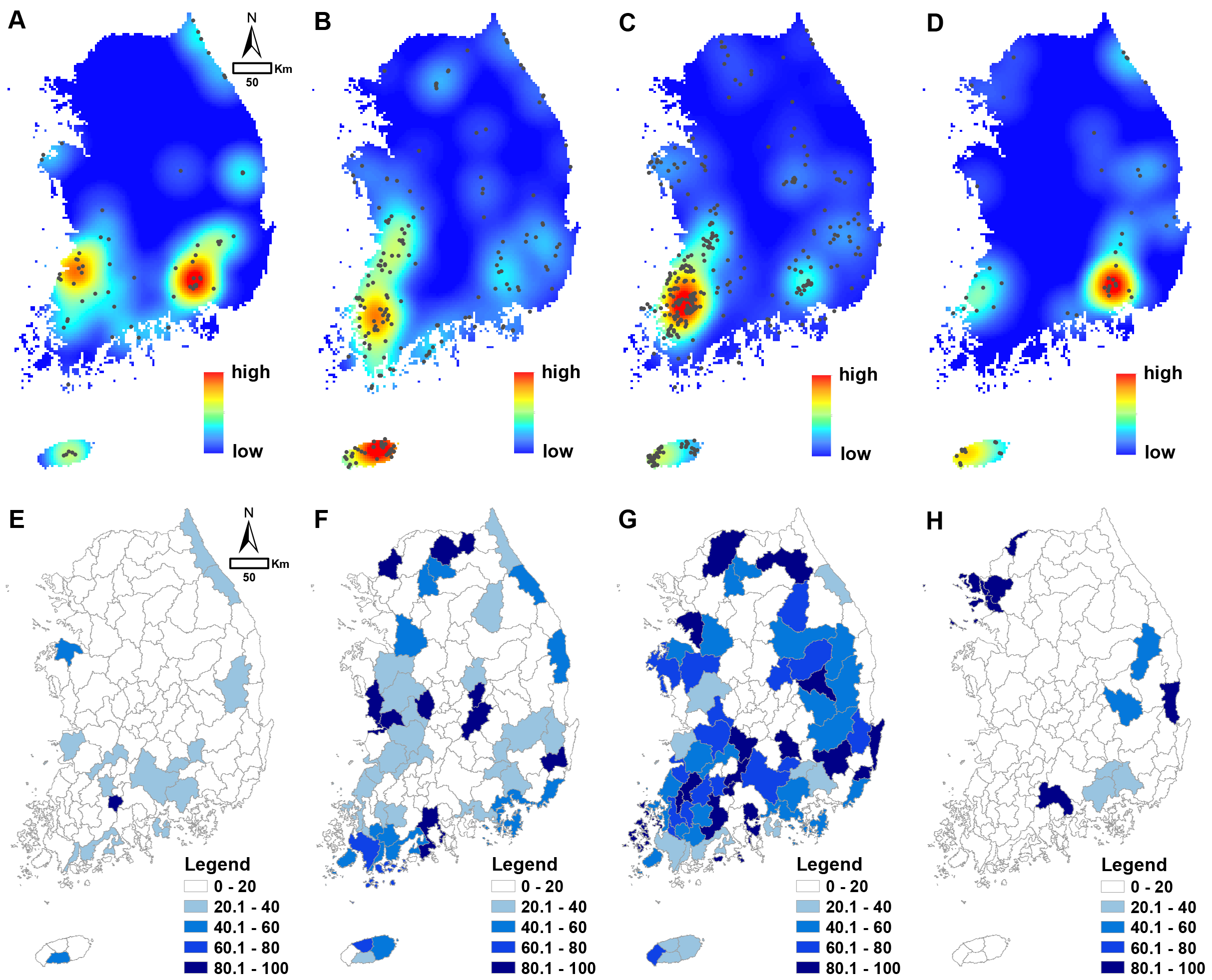

Supplement: Figure S2 — (A)–(D): point density lake-type wetlands with different ranks (A: A rank; B: B rank; C: C rank; D: D rank); (E)–(H): relative frequency of lake-type wetland rankings (%) in the catchment area (E: A rank; F: B rank; G: C rank; H: D rank) [file peerj-08-9101-s003.png]

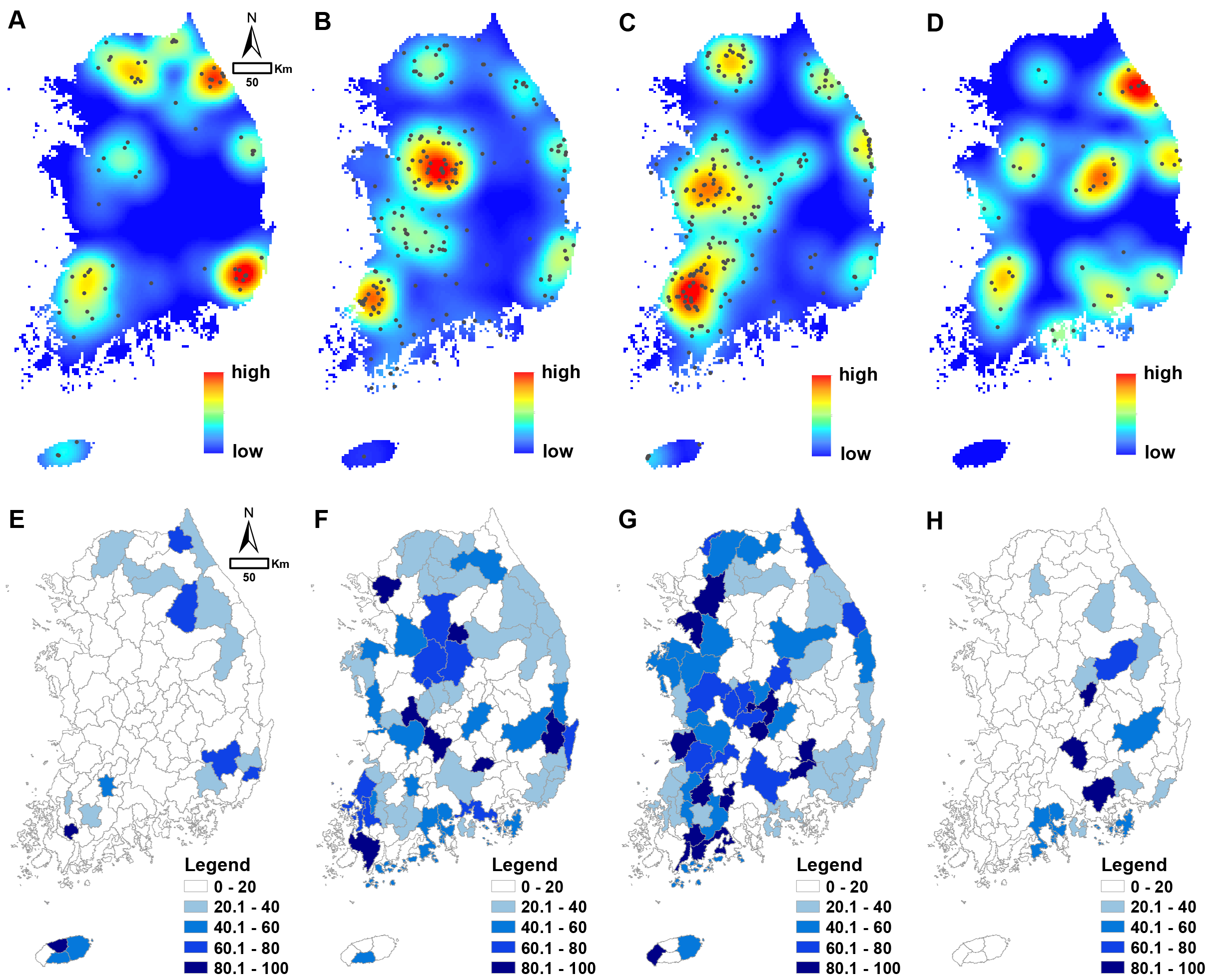

Supplement: Figure S3 — (A)–(D): point density of mountain-type wetlands with different ranks (A: A rank; B: B rank; C: C rank; D: D rank); (E)–(H): relative frequency of mountain-type wetland rankings (%) in the catchment area (E: A rank; F: B rank; G: C rank; H: D rank) [file peerj-08-9101-s004.png]

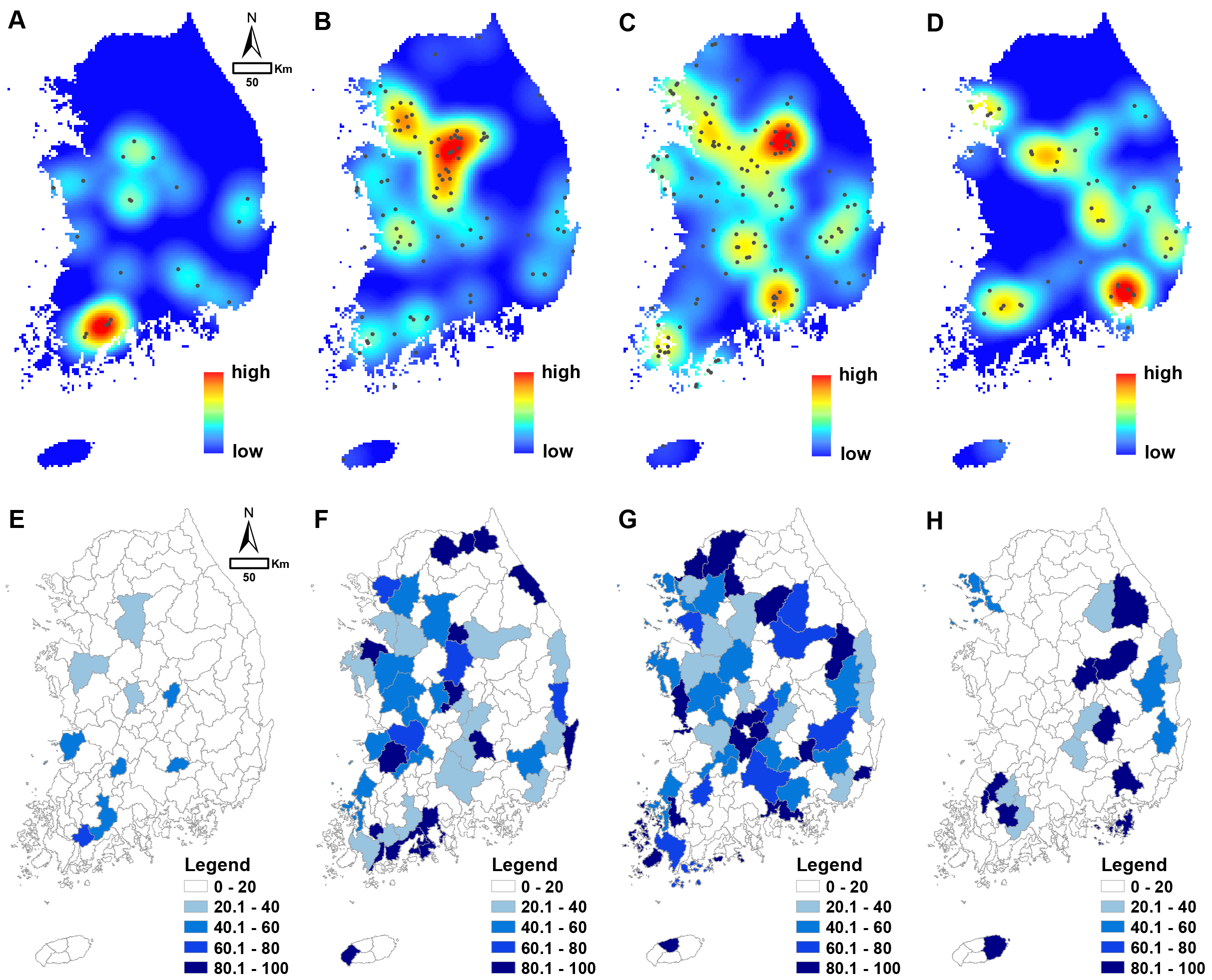

Supplement: Figure S4 — (A)–(D): point density of human-made wetlands with different ranks (A: A rank; B: B rank; C: C rank; D: D rank); (E)–(H): relative frequency of human-made wetland rankings (%) in the catchment area (E: A rank; F: B rank; G: C rank; H: D rank) [file peerj-08-9101-s005.png]
